# Urban tourist profiles during the pandemic in Taiwan: A multigroup analysis

**DOI:** 10.1016/j.heliyon.2023.e14157

**Published:** 2023-02-28

**Authors:** Sung-Ta Liu

**Affiliations:** Department of Urban Industrial Management and Marketing, University of Taipei, Taiwan

**Keywords:** Self-concordance, Tourism motivation, Tourism intention, Push and pull factors, Partial least squares structural equation modeling

## Abstract

Participating in tourism activities in crowded areas such as cities during the COVID-19 pandemic represents a risk. This study examined the demographic and psychological features of Taiwanese domestic urban tourists during the pandemic in 2021. The theoretical framework was based on push–pull motivation, self-concordance, and push–pull–mooring theories. The 680 valid questionnaire responses indicated that the respondents were generally interested in domestic urban tourism despite the pandemic threat. Moreover, 187 respondents regarded themselves as urban tourism seekers. Their demographic features were consistent with the typical primary urban tourism market profile: they were young, highly educated, and employed in skilled occupations. In terms of psychological features, the push factors, representing the individuals' intrinsic urban tourism motivations, were more potent than the pull factors, representing a city's tourism opportunities, as motivational drivers for increasing seekers' urban tourism intention during the pandemic. The methodology and findings of this study strengthen the literature on urban tourism and pandemic recovery.

## 1 Introduction

Urban tourism development is a multidisciplinary research area that includes urban planning, landscape planning, tourism planning and marketing, and destination branding [[1], [2], [3]]. Using the case of Taiwan, this research investigated the profiles of domestic urban tourism seekers and nonseekers during the COVID-19 pandemic. The examined variables were the two groups’ demographic profiles, internal and external motivations, and urban tourism intention during the pandemic.

Since late 2019, COVID-19 has diminished people's desire to engage in tourist activities and impaired the global tourism industry [[4], [5], [6]]. Notably, risks posed by the pandemic were higher in urban environments because of their greater population density [5,[7], [8], [9], [10]]. Some studies have discussed strategies for urban tourism recovery from the perspectives of policy-making, technology application, and event promotion [8,11,12]. Other studies have examined individuals' tourism preferences and intentions during the pandemic, with one example being a survey on urban tourism [13]. However, such studies have generally failed to address the essential factors related to individuals' intention to participate in urban tourism; these factors are especially pertinent because of the importance of tourist market segmentation [14]. Hence, the demographic and psychological characteristics of urban tourist segments during the pandemic warrant investigation.

Considering the aforementioned gap in the literature, this study incorporated push–pull motivation, self-concordance, and push–pull–mooring (PPM) theories into its theoretical framework. Empirical research has confirmed that tourism intention increases with motivation [15]. Some studies have classified tourism motivations into push and pull factors. These factors help clarify how individuals’ internal motivations influence their pursuit of psychological satisfaction through participation in tourist activities and how their external motivations influence their preference for specific activities [16,17].

Notably, although some studies have highlighted push factors as antecedent variables of pull factors, they have neglected to explain the mechanism underlying the relation [18,19]. Therefore, with reference to self-concordance theory, the study posited that push factors would have an enhancing effect on pull factors because the internal motivations of individuals increase their external motivations when pursuing a goal [[20], [21], [22], [23]].

Compared with intangible psychological features, tangible demographic factors are more readily observable indicators for tourism marketers to identify market segments [24,25]. Adopting a fast-paced urban lifestyle increases the need for recreational activities and leisure [26,27]. Therefore, studies have indicated that the target market for urban tourism is typically young, highly educated, skilled individuals [[28], [29], [30], [31], [32], [33]]. However, because these studies were conducted before the pandemic outbreak, an updated study considering the profiles of potential urban tourists in the pandemic context is necessary. Coincidentally, PPM theory suggests that individuals’ demographic characteristics can serve as a mooring factor that influences the effect of their push and pull motivations on intention [34,35]. Thus, demographic variables were included in the theoretical framework of this study.

Ultimately, the comprehensive theoretical framework adopted in this study allowed for comparing the demographic and psychological characteristics of two groups during the pandemic: urban tourism seekers and nonseekers. In addition to descriptive analysis, a chi-square test was used to identify the seekers’ demographic features. Moreover, partial least squares structural equation modeling (PLS-SEM) and PLS-based multigroup analysis (PLS-MGA) were used to compare the theoretical relationship between the two groups.

Following quota sampling, an online survey was conducted in Taiwan in April 2021. At the time, the pandemic prevention measures that Taiwan had implemented allowed its citizens to live relatively normal lives, in contrast to the reality in other countries [36]. Hence, the data collected in this study can be used to examine the promotion of urban tourism in the late pandemic period. Overall, this study contributes to the urban tourism literature and serves as a reference for urban tourism marketers involved in pandemic recovery efforts.

## 2 Literature review

On March 11, 2020, the World Health Organization officially declared COVID-19 a pandemic. Subsequently, countries began implementing epidemic-related prevention and control measures, such as border controls, lockdowns, and remote working. These measures strongly affected the global tourism industry. In contrast to natural disasters, pandemics usually do not damage tourism-related infrastructure; however, during a pandemic, people's tourism intention decreases because of concerns regarding viral infection, as indicated by survey studies [4,5] and studies conducting secondary data analysis [6].

In a positive sign for the tourism industry, a survey study by Wachyuni and Kusumaningrum (2020) revealed that individuals were positively disposed to participating in tourism activities once the pandemic weakened [13]. Additionally, studies in the tourism field have begun exploring whether individuals will be inclined to participate in tourist activities once they become accustomed to the “new normal” initiated by the pandemic and how their behavior may be altered. For instance, Zenker and Kock (2020) argued that tourists are likely to prefer short trips because of pandemic concerns [37]. Similarly, Miao et al. (2022) indicated that tourists might avoid overly popular tourist attractions and destinations [38]. In light of this complicated new context, studies have begun to consider how urban tourism might recover when pandemic diseases tend to spread rapidly in urban areas where social contact is frequent [5,[7], [8], [9], [10]].

Individuals who visit a city for leisure and recreation rather than for business, medical treatment, or other personal matters are considered general urban tourists [3]. Some studies have examined how urban tourism might recover from the adverse effect of COVID-19. For instance, McCartney et al. (2021) investigated how city authorities integrated urban tourism into a city's economic recovery [11], Casado-Aranda et al. (2021) suggested using technology to monitor visitors traveling in a city and trace potential virus carriers [8], and Perić et al. (2021) explored the hosting of public events in cities to attract tourists [12].

Although a crucial task of tourism marketers is to comprehend tourist market profiles [14], research on the features of urban tourists during the pandemic has been scant. Therefore, this study examined the demographic and psychological features of urban tourism seekers under the threat of COVID-19. In the following subsections, relevant literature is reviewed, and the development of the theoretical framework is described.

### 2.1 Urban tourism intention

Tourism scholars use the terms tourism/travel/visit intention to refer to an individual's willingness to participate in tourism activities, revisit the same locations for tourism-related purposes, and encourage others to do the same. Empirical studies have argued that tourism intention strongly predicts an individual's likelihood of visiting a certain location for tourism purposes [15,39,40]. Studies have thus investigated the factors that affect individuals' tourism intention in various contexts [[40], [41], [42], [43], [44]]. This study used urban tourism intention as the dependent variable to develop a theoretical framework for examining the demographic and psychological characteristics of urban tourists.

### 2.2 Urban tourism motivation (push and pull factors) and self-concordance

Tourism motivation refers to the intensity of an individual's desire to participate in tourist activities to satisfy their personal needs [45,46]. Tourism motivation is a widely recognized factor that positively influences tourism intention [15,45,46]. Researchers have employed diverse measurements to evaluate tourism motivation [3,47]. For instance, McIntosh and Goeldner (1990) designed a multidimensional leisure tourism motivation scale with physical, cultural, interpersonal, and status and prestige dimensions [48]; this scale has been adopted by other tourism-related empirical studies [49,50]. However, Goeldner modified the earlier approach and suggested that curiosity, relationships, and relaxation are three aspects of tourism motivation that fit broader study contexts [51].

Some studies have considered both push and pull factors as tourism motivations. Push factors refer to individuals’ internal motivations to participate in tourist activities (e.g., to escape daily life), whereas pull factors pertain to external motivations associated with the distinct characteristics of tourist destinations, attractions, and activities [17,52,53]. Initially proposed by Crompton (1979) [16], push–pull factors have been extensively conceptualized and empirically investigated in the tourism literature [17].

Arguably, push factors are similar to the aforementioned tourism motivation dimensions, as they focus mainly on attitudes, which are intangible [48,51]. By contrast, pull factors provide insight into how individuals assess the attractiveness of specific tourism venues [[54], [55], [56]]. Therefore, the measurement tool used for pull factors in empirical studies depends on the study subject, for example, a tourist destination, an attraction, or an activity [17,52,53]. Studies investigating the tourist attractions of cities have revealed several pull factors in urban tourism. For instance, Jansen-Verbeke (1986) suggested that a city's cultural, sports, and amusement facilities and physical and sociocultural features constitute its primary tourist attractiveness [57]; Law (2002) considered a city's heritage, national identity, famous people, and unique buildings as salient tourism attributes [3]; and Gau and Tu (2013) categorized the tourism characteristics of a city into three dimensions: space and facilities, environmental resources, and everyday life and culture [58].

Both push and pull factors exert a direct effect on tourism intention [53]. Several studies have demonstrated a correlation between the perception of push factors and the evaluation of pull factors [18,19]. However, none of these studies have clarified the theoretical basis for the relationship between push and pull factors. Therefore, to explain the relationship between the aforementioned variables, this study adopted self-concordance theory following a review of the contemporary theories of motivation.

According to self-concordance theory, individuals pursue goals for specific reasons, which can be classified as internal and external motivations. Individuals' internal motivations justify their external motivations. For example, an individual who is highly motivated to achieve a job promotion is expected to be aware of the company's incentive policy, whereas a less motivated individual is not [[20], [21], [22], [23]]. Therefore, on the basis of self-concordance theory, this study hypothesized that the internal urban tourism motivations (pull factors) of individuals justify their external motivations (pull factors) generated by the city's tourist attractiveness.

### 2.3 PPM theory and demographic features of urban tourism seekers

PPM theory, drawing on push and pull motivation research, emphasizes that certain antecedent variables, called mooring factors, can influence the effect of push and pull motivations on intention. Mooring factors can be individuals’ demographic features and subjective norms. PPM theory has been applied in tourism-related studies [34]. Although empirical studies have reported that some personal characteristics are not strong mooring factors, researchers would be wise not to overlook PPM theory and the possible influence of specific features on the relations between behavioral motivations and intentions [35].

In this study, the relevant literature was examined to identify mooring factors consistent with the study's objective and theoretical framework. The review indicated that individuals with different demographics have distinct concerns regarding COVID-19 [59]. Using individuals' contradictory attitudes regarding engaging in urban tourism during the pandemic as the mooring factor enabled this study (1) to compare the target demographic's motivations and intentions and (2) to identify the demographic features of individuals who consider themselves urban tourism seekers. Incidentally, from a practical management perspective, the demographic characteristics of specific tourist segments are more readily accessible than their psychological ones [24,25]. Before the COVID-19 outbreak, urban tourists tended to be young, highly educated, and skilled [[28], [29], [30], [31], [32], [33]]. Given the wide-reaching effects of the COVID-19 pandemic, novel empirical evidence regarding whether the pandemic promoted a shift in the primary market is necessary.

**Fig. 1:**
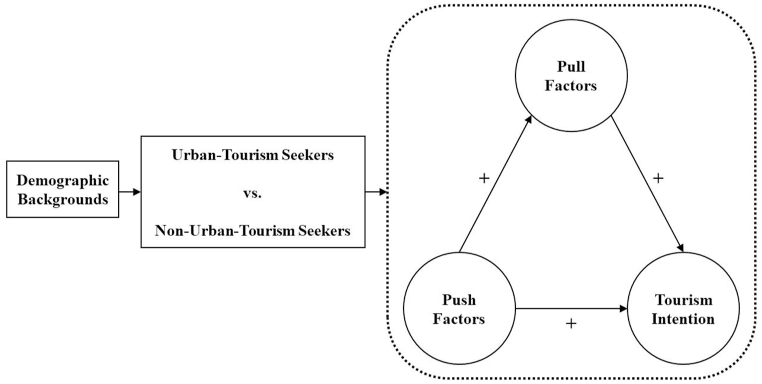
Conceptual model.

This study's theoretical framework was developed with reference to the preceding literature review (Fig. 1). The general demographic backgrounds of two groups, tourism seekers and nonseekers, with opposite attitudes toward urban tourism during the pandemic, were examined. The differences in these groups' attitudes toward urban tourism, which consist of the paths between push factors, pull factors, and tourism intention, were also investigated.

## 3 Methodology

Studies have suggested that in the aftermath of COVID-19, tourist preference would shift from long-haul trips to regional and domestic trips [37,38]; this study thus examined individuals’ domestic urban tourism motivations and intentions in Taiwan during the pandemic in 2021.

Taiwan is situated in the northwestern Pacific Ocean near other East Asian countries, including China to the northwest, Japan to the northeast, and the Philippines to the south. Because of the intense competition among global cities in the late 20th century, local governments of Taiwan have prioritized the development of urban tourism through initiatives such as regenerating urban spaces, implementing public relations campaigns on diverse media platforms, and hosting tourist events [36].

During the first half of 2021, when COVID-19 was at its peak in many countries, Taiwan's epidemic prevention performance earned a positive reputation; in Taiwan, life proceeded much as it had before the pandemic [37]; that is, despite the virus threat, intercity tourism was not severely affected [[60], [61]]. Additionally, Taiwan's small territory facilitates intercity mobility [62]. Because of the aforementioned factors, Taiwan is suitable for examining individuals' attitudes toward domestic urban tourism in the late pandemic era.

This study used a survey questionnaire for data collection. In the introduction to the questionnaire, the purpose of the study was outlined, and urban tourism was defined as visiting a domestic urban area for the purpose of engaging in leisure tourist activities instead of for medical or other personal reasons. In the questionnaire, the respondents provided their demographic information and identified themselves as urban tourism seekers or nonseekers during the pandemic. The respondents also expressed the strength of their urban tourism push motivations, pull motivations, and intentions when facing the pandemic threat. The participants rated each questionnaire item on a 7-point scale (1 = *completely disagree*, 7 = *completely agree*). The following sections provide details on the measurement models, statistical analysis techniques, and sampling method.

### 3.1 Measurement model design

Three measurement models based on the relevant literature were used to examine the psychological characteristics of the respondent with respect to domestic urban tourism: PUSH (push factors), PULL (pull factors), and INTENT (tourism intention). Appendix 1 presents the indicators in each measurement model, their respective reference sources, and related abbreviations.

The personnel responsible for survey distribution examined the phrasing of all items in the survey before formal data collection. All questionnaire items were presented in Mandarin. All PUSH indicators were formulated, whereas the PULL indicators were adapted from Gau and Tu (2003) and were also in Mandarin [59]. INTENT indicators IT1, IT2, and IT3 were from Chao and Lin's (2018) survey originally formulated in Mandarin [63]. Lastly, the IT4, IT5, IT6, and IT7 indicators, which were originally developed by Soundararajan and Singh (2019), were translated from English into Mandarin [43].

### 3.2 Statistical analysis techniques

SEM is used to evaluate the significance of relationships. Conventional covariance-based SEM is appropriate for processing reflective constructs, which comprise indicators related to each other. In this scenario, if one indicator of a reflective construct is deleted, then the construct's overall meaning remains unchanged. By contrast, deleting an indicator alters the meaning of the construct because a formative construct consists of independent indicators. Therefore, when formative constructs are analyzed, researchers must use PLS-SEM to avoid model misspecification and false statistical results [64,65].

Studies have typically treated intrinsic tourism motivations (push factors) and tourism intention as reflective constructs. However, for the pull-factor construct, each measurement indicator representing a city's particular tourist interest is independent of the other indicators [3,59]. Therefore, in this study, the pull-factor construct was regarded as formative, and PLS-SEM was used for relevant analyses.

Because this study compared urban tourism seekers with nonseekers, the sample sizes of both groups had to be sufficient to deliver statistical power. However, the respondents could only be categorized into two groups once they had completed the questionnaire. Given the maximum number of arrows pointing to a measurement model in this study was two, according to Hair et al. (2022), a total of 158 useable samples would be sufficient for PLS-SEM [65]. Therefore, in this study, the use of PLS-SEM enabled the collection of fewer samples without compromising the statistical robustness of the results.

In addition to the descriptive analysis, two segmentation approaches were adopted in this study to determine urban tourism seekers' demographic and psychological features: (1) a chi-square test was used to identify the statistical significance of the segments' demographic characteristics [24,25], and (2) PLS-MGA was used to consider significant differences between the two segments’ psychological features in terms of the paths within the same structural model assessed with data sets from each segment [65,66]. This study used SPSS 20 for Windows to conduct descriptive analysis, the chi-square test, and SmartPLS 3 for the PLS-SEM and PLS-MGA.

### 3.3 Sampling and data collection

Because of concerns regarding COVID-19 infection, data were gathered from an Internet panel offered by a private market survey company rather than through in-person surveys. On the bases of March 2021 census data from the Department of Household Registration of the Ministry of the Interior of Taiwan [67], quota sampling was conducted in April 2021, followed by data collection. A total of 680 valid questionnaires were collected. Although not included in the original quota sampling plan, 30 (4.4%) of the 680 respondents were older than 66. In Taiwan, adulthood is commonly accepted as beginning at 18, and the general retirement age is 65. Hence, individuals in the Internet panel aged 18–65 who could read Mandarin were eligible to participate in the survey. Appendix 2 presents the demographic information of the included respondents.

Regarding sample size, as previously mentioned, at least 158 useable samples for both urban tourism seekers and nonseekers were required to ensure statistical power. Among the 680 respondents, 187 and 493 self-identified as seekers and nonseekers during the pandemic. Therefore, the sample sizes of both groups were sufficient. Appendix 3 presents the groups’ demographic profiles.

## 4 Findings

### 4.1 Chi-square test

The chi-square test was used to identify significant relationships between the demographic characteristics and the compared segments. Among the four demographic variable sets, only age group and segment exhibited significant differences (p < .05; Appendix 4). Moreover, the crosstab results revealed that the numbers of urban tourism seekers in the 23–29 and 30–39 age groups were higher than expected. By contrast, the numbers of urban tourism seekers in the other age groups were lower than the expected counts (Appendix 5). This result suggests that the primary age range of urban tourists in Taiwan is 23–39 years. In Taiwan, people are usually engaged in undergraduate study until the age of approximately 22 years. This finding likely explains why the number of tourism seekers in this category was lower than expected.

Based on the aforementioned finding, the original age groups were recategorized into “23–39” and “other” to identify more detailed profiles of the markets. A chi-square test was conducted to compare the socioeconomic characteristics of these new age groups. The results indicated significant differences in occupation and education level (*p* < .05; Appendix 6). In addition, the crosstab results indicated that those in the “23–39” age group were more likely than those in the “other” age group to be skilled professionals and to have received a higher education; the actual sample counts in each category exceeded the corresponding expected counts (Appendix 7). The aforementioned findings are consistent with previous results suggesting that urban tourists are primarily youthful, skilled, and educated individuals [[28], [29], [30], [31], [32], [33]].

### 4.2 Mean scores of measurement items

All items had mean scores of >4.00 in all three data sets (overall, seekers, and nonseekers; Table 1). The results revealed that both seekers and nonseekers generally had positive intrinsic and external motivations and intentions related to urban tourism during the COVID-19 pandemic. The findings support related findings in the literature suggesting that urban tourism remained an option for individuals despite the pandemic threat [13].

**Table 1:** Mean scores of indicators.

Construct	Indicator	Overall (*N* = 680)		Seekers (*n* = 187)		Nonseekers (*n* = 493)	
		M	SD	M	SD	M	SD
PUSH	PS1	5.08	1.26	5.49	1.255	4.92	1.23
	PS2	4.80	1.32	5.18	1.203	4.66	1.34
	PS3	5.31	1.31	5.69	1.150	5.16	1.33
PULL	PL1	5.21	1.20	5.41	1.185	5.14	1.20
	PL2	5.46	1.18	5.62	1.266	5.40	1.14
	PL3	5.70	1.17	5.93	1.122	5.62	1.17
	PL4	5.14	1.17	5.38	1.178	5.05	1.15
	PL5	5.32	1.16	5.52	1.165	5.24	1.15
	PL6	5.31	1.14	5.50	1.184	5.24	1.12
	PL7	5.35	1.27	5.44	1.328	5.31	1.25
	PL8	5.37	1.17	5.67	1.076	5.26	1.19
	PL9	5.58	1.16	5.69	1.231	5.54	1.12
INTENT	IT1	4.78	1.46	5.32	1.365	4.57	1.45
	IT2	4.97	1.31	5.47	1.156	4.78	1.32
	IT3	4.72	1.34	5.23	1.289	4.53	1.30
	IT4	5.03	1.26	5.47	1.156	4.87	1.25
	IT5	5.05	1.30	5.48	1.228	4.88	1.29
	IT6	5.12	1.33	5.43	1.299	5.00	1.32
	IT7	5.13	1.29	5.60	1.138	4.95	1.30

### 4.3 PLS-SEM analysis of the overall data set

PLS-SEM analyses were conducted in two steps. In step 1, the relations between push factors, pull factors, and tourism intention were examined using three data sets (overall, seekers, and nonseekers) to evaluate the validity and reliability of the theoretical model in different contexts. In step 2, PLS-MGA was conducted to compare the paths in the structural models based on the seeker and nonseeker data sets. The data extracted during the PLS-SEM analyses were assessed according to the guidelines of Hair et al. (2022) [65].

In the model analysis of the overall data set, the outer loadings of the indicators of the two reflective constructs, PUSH and INTENT, exceeded 0.700, suggesting adequate indicator reliability. In addition, their composite reliability (CR) values exceeded 0.700, indicating consistent reliability, and their average variance extracted (AVE) values were greater than 0.500, indicating convergent validity. All reflective indicators were significant (*p* < .05; Table 2).

**Table 2:** Measurement model analysis for the overall data set (*N* = 680).

Construct and indicator	Outer weight/*p*	Outer loading/*p*	VIF	CR	AVE
PUSH				.912	.775
PS1	.416/.000	.908/.000	2.390		
PS2	.333/.000	.845/.000	1.925		
PS3	.384/.000	.885/.000	2.191		
PULL					
PL1	.349/.006	.783/.000	2.611		
PL2	.134/.246	.806/.000	3.649		
PL3	.242/.002	.732/.000	2.414		
PL4	.210/.013	.798/.000	2.527		
PL5	.276/.001	.831/.000	2.473		
PL6	.198/.011	.717/.000	2.289		
PL7	−.439/.000	.507/.000	2.635		
PL8	.132/.103	.724/.000	2.849		
PL9	.044/.611	.666/.000	2.763		
INTENT				.953	.743
IT1	.148/.000	.804/.000	2.565		
IT2	.170/.000	.887/.000	3.588		
IT3	.154/.000	.841/.000	2.883		
IT4	.171/.000	.891/.000	3.663		
IT5	.172/.000	.876/.000	3.537		
IT6	.164/.000	.826/.000	2.778		
IT7	.180/.000	.904/.000	4.016		

The variance inflation factor (VIF) values for all formative indicators within the PULL construct were less than 5.00, suggesting the absence of a high correlation between the indicators. The outer weights of all formative indicators were significant (*p* < .05) except those of PL2, PL8, and PL9. However, the outer loadings of PL2, PL8, and PL9 were greater than 500 and significant (*p* < .050). Hence, they were considered acceptable within the formative construct. Notably, the outer weight of PL7 was negative (−0.439), whereas those of the other eight formative indicators were positive, reflecting the nature of a formative construct with independent indicators (Table 2).

In the cross-factor loading analysis of the overall data set, the loading of each reflective indicator on its respective construct was greater than that on the other constructs, indicating discriminant validity (Appendix 8). Furthermore, the Fornell–Larcker criterion analysis of the overall data set revealed that the square root of the AVE for each reflective construct was higher than the correlation among any other construct pair, suggesting discriminant validity (Appendix 9).

To examine the relationship among the three constructs, 5000 bootstrapping iterations were performed (Table 3). All paths were significant (*p* < .05). Particularly, the PUSH→PULL and PUSH→INTENT paths had large effect sizes (*f*^2^ > 0.35), and the *f*^2^ of the PULL→INTENT path was .039, corresponding to small (0.02) and medium (0.15) effect sizes. Moreover, the PULL construct's coefficient of determination (*R*^2^) was 0.359, which was between 0.250 and 0.500, indicating low predictive accuracy. Furthermore, the *R*^2^ of the INTENT construct was 0.619, which was between 0.500 and 0.750, suggesting moderate predictive accuracy. A blindfolding method was adopted to estimate the Stone–Geisser *Q*^2^ values for dependent constructs and examine the structural model's predictive relevance. The *Q*^2^ value was >0.000, indicating predictive relevance. In addition, the structural model's standardized root-mean-square residual (SRMR) was 0.035, less than 0.080, indicating a favorable model fit [68].

**Table 3:** Structural model analysis results for the overall data set (*N* = 680).

Category	Path coefficient	*p*	*f*^2^	*R*^2^	*Q*^2^	SRMR
PUSH→PULL	.600	.000	.561			
PUSH→INTENT	.686	.000	.792			
PULL→INTENT	.151	.000	.039			
PULL				.359	.188	
INTENT				.619	.455	
Model fit						.035

The aforementioned statistical results for the overall data set supported the relations between the push factors, pull factors, and urban tourism intention, as indicated in the tourism literature [18,19] and suggested by self-concordance theory [[20], [21], [22], [23]].

In this study, because the model included a formative construct, the analysis procedure of Sarstedt et al. [68] was used to evaluate the robustness of the model in order to ensure the validity of the results by determining whether unobserved heterogeneity critically affects the results [69]. Finite mixture PLS was used for one to five segments, in which the default stop criterion was set to 1 × 10^−5^, the maximum number of iterations was set to 5,000, and the number of repetitions was set to 10. According to the results of fit indices for the one-to five-segment solutions, two types of Akaike's Information Criterion (AIC) indicated different segment numbers. In other words, modified AIC with factor 3 indicated a four-segment solution (criterion = 2675.974), whereas consistent AIC indicated a three-segment solution (criterion = 2773.155). Under these conditions, the three-segment solution seemed to be possible because it met the highest entropy statistic (normed) criterion (EN) (criterion = 0.588) (Appendix 10). However, because the three-segment solution did not meet the minimum sample size requirements for each segment, the two-segment solution was the only option (Appendix 11). In addition, the EN of the two-segment solution was 0.389 below 0.50, indicating that the unobserved heterogeneity was not at a critical level, thereby supporting analysis results of the overall data set (Appendix 10).

### 4.4 PLS-SEM analysis of the urban tourism seeker data set

The same analysis techniques described earlier were adopted for the tourism seeker data set; the results indicated that the theoretical framework also fit the seeker data set. The results were similar to those obtained for the overall data set. Notably, the outer weight of item PL7 was also negative (−0.076; Table 4, Table 5 and Appendices 12 and 13).

**Table 4:** Measurement model analysis results for the urban tourism seeker data set (*n* = 187).

Construct and indicator	Outer weight/*p*	Outer loading/*p*	VIF	CR	AVE
PUSH				.893	.736
PS1	.423/.000	.891/.000	2.089		
PS2	.367/.000	.855/.000	1.933		
PS3	.374/.000	.827/.000	1.652		
PULL					
PL1	.365/.004	.871/.000	3.392		
PL2	.070/.718	.835/.000	4.345		
PL3	.256/.048	.791/.000	2.162		
PL4	.042/.753	.767/.000	2.739		
PL5	.151/.196	.767/.000	2.593		
PL6	.284/.014	.806/.000	2.124		
PL7	−.076/.563	.710/.000	2.451		
PL8	.103/.328	.740/.000	2.466		
PL9	.030/.858	.720/.000	2.949		
INTENT				.932	.663
IT1	.161/.000	.738/.000	2.132		
IT2	.184/.000	.841/.000	2.618		
IT3	.155/.000	.804/.000	2.378		
IT4	.183/.000	.844/.000	2.713		
IT5	.179/.000	.824/.000	2.664		
IT6	.166/.000	.780/.000	2.419		
IT7	.198/.000	.862/.000	2.879

**Table 5:** Structural model analysis results for the urban tourism seeker data set (*n* = 187).

Category	Path coefficient	*p*	*f*^2^	*R*^2^	*Q*^2^	SRMR
PUSH→PULL	.614	.000	.606			
PUSH→INTENT	.518	.000	.507			
PULL→INTENT	.392	.000	.291			
PULL				.377	.208	
INTENT				.671	.434	
Model fit						.048

### 4.5 PLS-SEM analysis of the nonseeker data set

Using the same techniques described previously, this study examined the nonseeker data and discovered that the data set fit the structural model. As with the analyses of the other two data sets, the outer weight of item PL7 was negative (−0.569) (Table 6, Table 7 and Appendices 14 and 15).

**Table 6:** Measurement model analysis results for the nonseeker data set (*n* = 493).

Construct and indicator	Outer weight/*p*	Outer loading/*p*	VIF	CR	AVE
PUSH				.913	.777
PS1	.419/.000	.912/.000	2.470		
PS2	.323/.000	.833/.000	1.855		
PS3	.388/.000	.898/.000	2.383		
PULL					
PL1	.357/.025	.731/.000	2.412		
PL2	.206/.147	.785/.000	3.623		
PL3	.157/.145	.678/.000	2.697		
PL4	.253/.015	.780/.000	2.434		
PL5	.334/.001	.834/.000	2.424		
PL6	.160/.119	.654/.000	2.405		
PL7	−.569/.000	.421/.000	2.768		
PL8	.087/.425	.676/.000	3.096		
PL9	.110/.265	.641/.000	2.841		
INTENT				.956	.755
IT1	.141/.000	.810/.000	2.634		
IT2	.169/.000	.892/.000	3.836		
IT3	.153/.000	.842/.000	2.919		
IT4	.168/.000	.898/.000	4.105		
IT5	.173/.000	.886/.000	3.843		
IT6	.167/.000	.839/.000	3.040		
IT7	.178/.000	.910/.000	4.425

**Table 7:** Structural model analysis results for the nonseeker data set (*n* = 493).

Category	Path coefficient	*p*	*f*^2^	*R*^2^	*Q*^2^	SRMR
PUSH→PULL	.590	.000	.533			
PUSH→INTENT	.710	.000	.821			
PULL→INTENT	.103	.010	.017			
PULL				.348	.208	
INTENT				.600	.434	
Model fit						.038

### 4.6 PLS-MGA of urban tourism seekers and nonseekers

The theoretical framework formulated in this study fit the overall data set and the data sets of the two groups. Therefore, PLS-MGA was used to identify significant differences between the paths in the model based on the two groups’ data sets.

The PLS-MGA analysis revealed significant differences in the path coefficients of PUSH→INTENT and PULL→INTENT (*p* < .05). Moreover, the negative coefficients of the PUSH→INTENT path indicated that the influence of intrinsic motivations on tourism intention for seekers was not as strong as for nonseekers. Additionally, the difference in the path coefficients of PULL→INTENT was positive, indicating that the influence of external motivations on tourism intention was more substantial for seekers than nonseekers (Table 8).

**Table 8:** MGA results.

Path	Path coefficients difference (Seekers–Nonseekers)	*t*	*p*
PUSH→PULL	0.025	0.366	0.694
PUSH→INTENT	−0.192	2.375	0.034
PULL→INTENT	0.289	3.596	0.001

## 5 Discussion

The descriptive analysis indicated that 187 of the 680 respondents considered themselves domestic urban tourists during the pandemic, whereas 493 did not. Although the sample size of urban tourism seekers was considerably smaller than that of nonseekers, both groups had positive urban tourism motivations and intentions. Therefore, despite urban areas presenting a relatively high risk of COVID-19 infection [5,[7], [8], [9], [10]], Taiwanese cities remained a tourism option for both groups.

The results also indicated that the urban tourism seekers in this study were predominantly aged between 23 and 39 years, had completed higher education and had skilled occupations in tertiary industries. This market profile is in line with typical urban tourists, as suggested in the literature [[28], [29], [30], [31], [32], [33]].

The aforementioned results are consistent with studies suggesting that the pandemic affected individuals’ perception of risk but not tourism infrastructure [[4], [5], [6],^70^] and that individuals maintained a positive attitude toward tourism recovery [13,39]. Therefore, during the COVID-19 pandemic, the primary urban tourist market did not exhibit a substantial shift at the domestic level.

Because relevant studies have not explained why push factors affect pull factors [18,19], self-concordance theory [[20], [21], [22], [23]] was adopted in this study to determine the relations between push and pull factors. The PLS-SEM results confirmed all of the relations regardless of whether the overall, seeker, or nonseeker data sets were used to test the theoretical framework; consistently, the respondents' urban tourism motivations positively affected their urban tourism intentions. More importantly, their autonomous motivations (push factors) enhanced the effect of external motivations (pull factors). Hence, domestic urban tourism marketers can consider strengthening individuals’ autonomous urban tourism motivations as a strategic priority.

Notably, as was the case for the other eight formative indicators of the pull-factor construct, item PL7 (highly commercialized environments) had a mean score higher than the scale's midpoint in all the data sets. However, PL7 was the only formative item with a negative indicator weight in the PLS-SEM analyses. This finding can be attributed to the nature of formative constructs; the indicators were independent [64,65]. Moreover, from a managerial perspective, highly commercialized environments are widely recognized urban tourism elements with negligible impact on tourists.

The PLS-MGA revealed that the samples' interest in urban tourism had no effect on the strength of the relationship between internal and external motivation. However, the effect of intrinsic motivations on urban tourism intention was less prominent among urban tourism seekers than among nonseekers. By contrast, the effect of external motivations on tourism intention was more salient among seekers than nonseekers. These significant differences may be attributable to seekers already having established internal motivations. This finding is consistent with another study on PPM theory that suggested that individuals’ characteristics might generate differences in the relations between their motivations and intentions [34]. This finding may serve as a reference for domestic Taiwanese urban tourism marketers developing recovery strategies.

## 6 Conclusion

This study compared the demographic profiles, motivations, and intentions of domestic urban tourism seekers and nonseekers in Taiwan in 2021, a scenario reflecting the late pandemic period [37]. This empirical work yielded five key findings providing insights into the recovery of the tourism industry based on changes in tourist behavior instead of policy-setting conclusions [8,11,12].

First, even though the risk of infection during pandemics is elevated in urban areas, Taiwanese cities remained a tourism option for the inbound market.

Second, the majority of urban tourism seekers in this study were between ages of 23 and 39 years, held advanced degrees, and worked in skilled tertiary industries. Therefore, the pandemic had no effect on the general profile of primary urban tourism market segments.

Third, the theoretical framework based on push–pull factors and self-concordance theory revealed that internal urban tourism motivations increased external urban tourism motivations. Both motivations increased urban tourism intention, which was true regardless of whether the data sets were collected from urban tourism seekers or nonseekers.

Fourth, consistent with the notion in PPM theory that individuals’ characteristics result in differences between the influence of their motivations on intentions, pull and push factors were more effective incentives for seekers and nonseekers, respectively. Thus, marketers should increase the intentions of urban tourism seekers by stimulating their external motivations and reinforcing the intrinsic motivations of nonseekers.

Fifth, the use of a formative model with independent measurements within the theoretical framework revealed that highly commercialized environments may not be a salient selling point of urban destinations.

This study has some limitations. First, Taiwan was selected as the study setting. Future studies may consider collecting data from respondents in other cultural contexts. Second, some survey questions were drawn from studies whose respondents were Mandarin speakers. In the future, researchers may consider adopting different measurement indicators from a broader literature base. Third, the demographic and psychological characteristics of domestic urban tourism respondents were investigated. Further research can expand on this theme to investigate other urban tourism markets, such as inbound and outbound markets. Finally, with reference to PPM theory, this study compared the psychological features of urban tourism seekers and nonseekers. Future studies may consider whether other variables constrain or moderate the effects of push and pull factors in urban tourism.

## Author contribution statement

Sung-Ta Liu, PhD: Conceived and designed the experiments; Performed the experiments; Analyzed and interpreted the data; Contributed reagents, materials, analysis tools or data; Wrote the paper.

## Funding statement

Dr Sung-Ta Liu was supported by Ministry of Science and Technology, Taiwan [109-2410-H-128-028-].

## Data availability statement

Data will be made available on request.

## Declaration of interest's statement

The author declares no conflict of interest.

## Figures and Tables

**Table:** 

Construct	Item
PUSH (push factors)	PS1: I choose urban areas for leisure tourism because it arouses and satisfies my curiosity [52].
	PS2: I choose urban areas for leisure tourism because it encourages and satisfies my desire to build connections and interact with others [52].
	PS3: I choose urban areas for leisure tourism because it satisfies my desire to relax [52].
PULL (pull factors)	PL1: Urban tourism provides the experience of modern space design [59].
	PL2: Urban tourism provides high-quality public services and facilities [3,59].
	PL3: Urban tourism provides convenient public transportation [3,59].
	PL4: Urban tourism provides an opportunity to view beautiful human-made landscapes [3,59].
	PL5: Urban tourism provides an opportunity to visit unique landmarks [3,59].
	PL6: Urban tourism provides an opportunity to attend various artistic events [3,59].
	PL7: Urban tourism provides access to highly commercialized environments [3,59].
	PL8: Urban tourism provides an opportunity to engage in various entertainment activities [3,59].
	PL9: Urban tourism provides an opportunity to access satisfying shopping environments [3,59].
INTENT (tourism intention)	IT1: When budgeting is a concern, I visit urban areas for leisure tourism [63].
	IT2: When searching for tourism information, I tend to spend more time considering urban areas [63].
	IT3: I recommend people in my social groups visit urban areas for leisure tourism [63].
	IT4: Generally, I am interested in urban tourism [43].
	IT5: Visiting urban areas for leisure tourism is relevant to me [43].
	IT6: Planning urban tourism activities is relevant to me [43].
	IT7: I am interested when others tell me about urban tourism [43].

**Table:** 

Category	Variable	Population	Quota		True	
			Ratio (%)	*N*	Ratio (%)	*N*
Sex	Male	8,191,408	49.9	299	49.9	339
	Female	8,225,065	50.1	301	50.1	341
	Total	16,416,473	100.0	600	100.0	680
Age (years)	18–22	1,342,794	8.2	49	7.8	53
	23–29	2,229,593	13.6	81	12.9	88
	30–39	3,450,443	21.0	126	20.2	137
	40–49	3,779,389	23.0	138	22.1	150
	50–59	3,621,539	22.1	132	21.0	143
	60–65	1,992,715	12.1	73	11.6	79
	Over 66				4.4	30
	Total	16,416,473	100.0	600	100.0	680
Location in Taiwan	North	7,843,719	47.9	287	48.1	327
	Central	3,657,817	22.3	134	22.3	152
	South	4,348,885	26.5	159	26.5	180
	East and others	566,052	3.5	20	3.1	21
	Total	16,416,473	100.0	600	100.0	680

**Table:** 

Category	Variable	Seekers		Nonseekers	
		Ratio (%)	*n*	Ratio (%)	*n*
Sex	Male	49.7	93	49.9	246
	Female	50.3	94	50.1	247
	Total	100.0	187	100.0	493
Age (years)	18–22	5.9	11	8.5	42
	23–29	16.0	30	11.8	58
	30–39	25.1	47	18.3	90
	40–49	20.3	98	22.7	112
	50–59	21.9	41	20.7	102
	Over 60	10.7	20	18.1	89
	Total	100.0	187	100.0	493
Occupation	Primary and secondary industries	22.5	42	16.6	82
	Tertiary industry	34.2	64	40.6	200
	Public servant	10.7	20	7.7	38
	Students	4.8	9	6.1	30
	Retirement/Housemaker/Unemployment/others	27.8	52	29.0	143
	Total	100.0	187	100.0	493
Education	Non-higher education	24.6	46	24.1	119
	Undergraduate	66.8	125	67.1	331
	Postgraduate	8.6	16	8.7	43
	Total	100.0	187	100.0	493

**Table:** 

Variable	Pearson Chi-square	*Df*	*P*
Sex	.001	1	.969
Age (years)	11.341	5	.045
Occupation	5.799	4	.215
Education	.018	2	.991

**Table:** 

Age (years)	Seekers (*n* = 187)		Nonseekers (*n* = 493)	
	Count	Expected	Count	Expected
18–22	11	14.6	42	38.4
23–29	30	24.2	58	63.8
30–39	47	37.7	90	99.3
40–49	38	41.3	112	108.8
50–59	41	39.3	102	103.7
Over 60	20	30.0	89	79.0

**Table:** 

Variable	Pearson Chi-square	*Df*	*p*
Occupation	59.114	4	.000
Education	36.381	2	.000

**Table:** 

Category	Variable	23–39 (*n* = 225)		Others (*n* = 455)	
		Count	Expected	Count	Expected
Occupation	Primary and secondary industries	44	41.0	80	83.0
	Tertiary industry	110	87.4	154	176.6
	Public servant	34	19.2	24	38.8
	Students	3	12.9	36	26.1
	Retirement/Housemaker/Unemployment/others	34	64.5	161	130.5
Education	Non-higher education	23	54.6	142	110.4
	Undergraduate	177	150.9	279	305.1
	Postgraduate	25	19.5	34	39.5

**Table:** 

Indicator	PUSH	INTENT
PS1	.908	.768
PS2	.845	.582
PS3	.885	.686
IT1	.602	.804
IT2	.693	.887
IT3	.618	.841
IT4	.688	.891
IT5	.690	.876
IT6	.663	.826
IT7	.725	.904

**Table:** 

Construct	PUSH	INTENT
PUSH	.880	
INTENT	.777	.862

**Table:** 

Criteria	Number of segments				
	1	2	3	4	5
Modified AIC with Factor 3	2915.834	2732.376	2696.279	2675.974	2682.385
Consistent AIC	2938.444	2782.119	2773.155	2779.982	2813.526
Entropy Statistic (Normed)		.389	.558	.472	.456

**Table:** 

Number of segments	Segment 1	Segment 2	Segment 3	Segment 4	Segment 5
1	1				
2	.623	.377			
3	.614	.311	.075		
4	.517	.239	.17	.075	
5	.500	.167	.145	.113	.075

**Table:** 

Indicator	PUSH	INTENT
PS1	.891	.721
PS2	.855	.602
PS3	.827	.623
IT1	.605	.738
IT2	.647	.841
IT3	.544	.804
IT4	.660	.844
IT5	.590	.824
IT6	.579	.780
IT7	.686	.862

**Table:** 

Construct	PUSH	INTENT
PUSH	.858	
INTENT	.759	.814

**Table:** 

Indicator	PUSH	INTENT
PS1	.912	.772
PS2	.833	.555
PS3	.898	.688
IT1	.576	.810
IT2	.688	.892
IT3	.618	.842
IT4	.681	.898
IT5	.704	.886
IT6	.681	.839
IT7	.721	.910

**Table:** 

Construct	PUSH	INTENT
PUSH	.882	
INTENT	.770	.869

## Appendix

Appendix 1. Main survey items of measurement models.

Appendix 2. Respondents’ demographic information (*N* = 680).

Appendix 3. Demographic profiles of two segments (*N* = 680).

Appendix 4. Chi-square test results for seekers and nonseekers (*N* = 680).

Appendix 5. Crosstab of age and segment variables (*N* = 680).

Appendix 6. Chi-square test results for new age groups (*N* = 680).

Appendix 7. Crosstab of the occupation and education of new age groups (*N* = 680).

Appendix 8. Cross-loading analysis results for the overall data set (*N* = 680).

Appendix 9. Fornell–Larcker criterion analysis results for the overall data set (*N* = 680).

Appendix 10. Fit indices for the one-to five-segment solutions (*N* = 680).

Appendix 11. Relative segment sizes (*N* = 680).

Appendix 12. Cross-loading analysis results for the urban tourism seeker data set (*n* = 187).

Appendix 13. Fornell–Larcker criterion analysis results for the urban tourism seeker data set (*n* = 187).

Appendix 14. Cross-loading analysis results for the nonseeker data set (*n* = 493).

Appendix 15. Fornell–Larcker criterion analysis results for the nonseeker data set (*n* = 493).
