# Supplementary material for: Urban tourist profiles during the pandemic in Taiwan: A multigroup analysis
Source: Heliyon. 2023 Feb 28;9(3):e14157. doi: 10.1016/j.heliyon.2023.e14157 (PMC10006644; doi:10.1016/j.heliyon.2023.e14157)
Supplement: Multimedia component 1 [file mmc1.pdf]

(The study used an Internet panel offered by a private market survey company, rather than an in-person survey format, for data collection. The arrangement was addressed in section *3.3. Sampling and data collection* in the manuscript. The study did not include clinical research and human subjects. The questionnaire did not acquire the respondents' sensitive personal data. The respondents were informed their answers were used for academic purposes only.)

Please rate the degree to which you agree or disagree with each of the following statements (1 = *completely disagree*, 2 = *mostly disagree*, 3 = *slightly disagree*, 4 = *neither agree nor disagree*, 5 = *slightly agree*, 6 = *mostly agree*, 7 = *completely agree*).

- Intrinsic motivations:
  - I choose urban areas for leisure tourism because it arouses and satisfies my curiosity.
  - I choose urban areas for leisure tourism because it encourages and satisfies my desire to build connections and interact with others.
  - I choose urban areas for leisure tourism because it satisfies my desire to relax.

- External motivations:
  - Urban tourism provides the experience of modern space design.
  - Urban tourism provides high-quality public services and facilities
  - Urban tourism provides convenient public transportation.
  - Urban tourism provides an opportunity to view beautiful human-made landscapes.
  - Urban tourism provides an opportunity to visit unique landmarks.
  - Urban tourism provides an opportunity to attend various artistic events.
  - Urban tourism provides access to highly commercialized environments.
  - Urban tourism provides an opportunity to engage in various entertainment activities.
  - Urban tourism provides an opportunity to access satisfying shopping environments.

- Intention:
  - When budgeting is a concern, I visit urban areas for leisure tourism.
  - When searching for tourism information, I tend to spend more time considering urban areas.

- I recommend people in my social groups to visit urban areas for leisure tourism.
- Generally, I am interested in urban tourism.
- Visiting urban areas for leisure tourism is relevant to me.
- Planning urban tourism activities is relevant to me.
- I am interested when others tell me about urban tourism.

Please choose the answer that fits you best.

- Your sex:
  - Male
  - Female
  - Prefer not to say

- Your age (years):
  - 18–22
  - 23–29
  - 30–39
  - 40–49
  - 50–59
  - 60–65
  - Over 66

- Your occupation:
  - Primary and secondary industries
  - Tertiary industry
  - Public servant
  - Students
  - Retirement/Housemaker/Unemployment/Others

- Your education:
  - Non-higher education
  - Undergraduate
  - Postgraduate

- Your residency location in Taiwan:
  - North
  - Central

- South
- East and others

- Whether you miss urban tourism after the outbreak of the COVID-19 pandemic?
  - Yes
  - No
